# CXCL10 Chemokine: A Critical Player in RNA and DNA Viral Infections

**DOI:** 10.3390/v14112445

**Published:** 2022-11-03

**Authors:** Noha Mousaad Elemam, Iman Mamdouh Talaat, Azzam A. Maghazachi

**Affiliations:** 1 Department of Clinical Sciences, College of Medicine, University of Sharjah, Sharjah 27272, United Arab Emirates; 2 Sharjah Institute for Medical Research, University of Sharjah, Sharjah 27272, United Arab Emirates; 3 Pathology Department, Faculty of Medicine, Alexandria University, Alexandria 21131, Egypt

**Keywords:** chemokines, RNA viruses, DNA viruses, CXCL10

## Abstract

Chemokines constitute a group of small, secreted proteins that regulate leukocyte migration and contribute to their activation. Chemokines are crucial inflammatory mediators that play a key role in managing viral infections, during which the profile of chemokine expression helps shape the immune response and regulate viral clearance, improving clinical outcome. In particular, the chemokine ligand CXCL10 and its receptor CXCR3 were explored in a plethora of RNA and DNA viral infections. In this review, we highlight the expression profile and role of the CXCL10/CXCR3 axis in the host defense against a variety of RNA and DNA viral infections. We also discuss the interactions among viruses and host cells that trigger CXCL10 expression, as well as the signaling cascades induced in CXCR3 positive cells.

## 1. Role of Chemokines during Viral Infections

Chemokines are small, secreted molecules that enhance cell interaction via G-protein-coupled receptors [1]. The chemokine-encoding genes in humans are located on chromosomes 4 and 17 [2]. In addition to being active as monomers, during an immune response, chemokines form homodimers and heterodimers, which result in a robust immunological response [3,4]. A general classification of chemokines is based on the structure of the two cysteine residues closest to the N terminus, which can be juxtaposed (CC) or separated by a single amino acid (CXC) or by three amino acids (CX3C). Chemokines that lack the first cysteine residue are known as XC chemokines [5,6]. Others classified chemokines into two primary categories according to their functions: those engaged in homeostasis and those involved in inflammation. Chemokine biological effects include many processes, such as angiogenesis regulation, tumor development, and invasion [7].

Further, chemokine receptors can be categorized into conventional (cCKRs) and atypical [8]. Conventional receptors can heterodimerize with other cCKRs, membrane proteins, and atypical chemokine receptors in addition to existing as homodimers and oligomers [9,10]. It should be noted that chemokines may also operate as natural antagonists rather than inducing activating signals when they attach to their appropriate receptors [11]. On the other hand, atypical chemokine receptors (ACKRs) are structurally linked to cCKRs when they bind to chemokines with a high affinity. However, because the intracellular signaling motifs are absent or altered, they do not activate the same signaling pathways as cCKRs [12,13]. In other words, the perturbation of ACKRs does not activate the G-protein signaling pathway but instead internalizes the chemokines to be degraded through the recruitment of β-arrestins [8]. Functionally, atypical chemokine receptors are known to regulate the localization, distribution, and abundance of chemokines and are considered proficient scavengers, thus modulating chemokine concentrations and bioavailability [14]. Moreover, ACKRs prevent unnecessary desensitization upon excessive exposure to chemokine ligands [15,16,17,18].

Several physiological processes, including cell proliferation and angiogenesis, as well as pathological conditions, including inflammation, neoplasia, autoimmunity, and infections (viral, bacterial, or parasitic), are controlled by binding chemokines to their cognate receptors [19]. During viral infections, chemokines are released as a result of a series of events [20], connecting several components [21] that boost the innate and adaptive immune systems which promote cell migration towards the sites of infection [22]. Nevertheless, viruses attempt to elude the immune system by molecular mimicry [23].

## 2. Structure and Function of CXCL10

Three chemokines are known to be interferon (IFN)-induced angiostatic CXC chemokines. These include monokine induced by interferon (MIG/CXCL9), interferon gamma-induced protein 10 (IP-10/CXCL10), and interferon-inducible T-cell chemoattractant (I-TAC/CXCL11). The chemokine CXCL10 binds CXCR3, which is present in T helper lymphocytes, natural killer (NK) cells, dendritic cells (DCs), macrophages, and B cells [24,25,26,27]. CXCR3 has two different isoforms, CXCR3-A and CXCR3-B, that possess several functions. For example, upon the binding of CXCL10 to isoform A, chemotaxis and proliferation are induced [28,29], while binding to the B isoform results in inhibiting cell migration and proliferation [29,30]. The atypical chemokine receptor ACKR2 is known to bind most inflammatory CC chemokine members [31] and is activated by the agonist CXCL10. Chevigné et al. described a novel aspect of CXCL10 regulation [32]. In an inflammatory status, CXCL10 could be secreted by various immune cells such as neutrophils, eosinophils, and monocytes, as well as epithelial cells, endothelial cells, and fibroblasts upon IFN-γ triggering [33,34,35]. Consequently, activated Th1 lymphocytes, monocytes, and NK cells would migrate towards the sites of inflammation in order to trigger the release of pro-inflammatory cytokines and chemokines. CXCL10 does not have the structural domain ELR “Glu-Leu-Arg” tripeptide motif, which is present in certain CXC chemokines and which might have anti-angiogenic along with anti-tumor properties [30,36,37,38,39]. The gene coding for CXCL10 is localized on chromosome 4 at band q21, and its translation produces a 12 kDa protein that has 2 internal disulfide cross-bridges [34]. Upon cleavage, a 10 kDa protein is generated and secreted with 4 conserved cysteine residues in the N-terminal [34]. The structure of CXCL10 exhibits a typical chemokine fold consisting of a three-stranded β sheet overlaid by an α helix with a number of the receptor binding residues located in the associated loops stabilized by the disulfide bonds [40]. The transcription of CXCL10 is regulated by various external stimuli, including cytokines such as IFN-γ and bacterial components such as lipopolysaccharides (LPS). 

CXCL10 possesses an upstream regulatory sequence that contains several critical regulatory elements for nuclear factor-κB (NF-κB) and interferon-stimulated response element (ISRE), as well as sites for the binding of proteins such as heat shock (HS) factors [41]. Cytokines such as tumor necrosis factor (TNF-α) and IFN-γ induce the expression of CXCL10 through NF-κB and ISRE present in the promoter of CXCL10 [42,43,44]. This could lead to the activation of protein kinase C and increase the intracellular Ca^2+^ mobilization [45,46]. Since it is an inflammatory chemokine, CXCL10 was associated with multiple disorders, including infectious diseases, autoimmune diseases, and cancer [18,47,48,49,50]. Furthermore, it exacerbates inflammation, causing tissue damage [47]. Moreover, CXCL10 was expressed within tissues following viral infections, suggesting an essential role for this chemokine in host defense by contributing to the lymphocyte activation, migration, and infiltration of specific T cell subsets within the sites of infection [51]. 

## 3. Role of CXCL10 in RNA Viral Infections

CXCL10 could be protective or pathogenic in viral infections depending on the host’s immune status and the type of viral infection [52]. CXCL10 has been implicated in multiple RNA viral infections, including rhinovirus, respiratory syncytial virus (RSV), coxsackie virus, hepatitis C virus (HCV), Ebola, dengue, and equine infectious anemia virus [33,53,54,55,56,57,58,59,60]. It is also involved in various models of respiratory viral infection, including Sendai, influenza, corona, and adenoviruses, as well as neurogenic viral reactions where the expression and role of CXCL10 have been reported [61,62,63,64,65,66]. This highlights the significance of CXCL10 expression in multiple viral infections that could aid in viral clearance or contribute to disease pathogenesis.

The absence of CXCL10 using antibodies or knockout mouse models resulted in increased viral titers and reduced T cell infiltration within the brains of mice infected with a murine hepatitis virus (MHV), leading to increased mortality [67,68]. CXCL10 also affects the migration of CD8^+^ T cells in the liver of adenovirus infected mice [69,70]. These studies indicate that CXCL10 is crucial in host defense and the development of a protective T cell response during viral infections. Furthermore, CXCL10 has been shown to induce the migration of T cells and NK cells following viral infections [71,72,73,74], where it attracts NK cells into the CNS post MHV infection [51]. Moreover, CXCL10 limits the spread of infection, contributing to early host defense and associated with a direct anti-viral effect against several RNA viruses [75,76]. For instance, CXCL10 blocked the entry and replication of the dengue virus by inhibiting its binding to the cell surface receptors [77]. Additionally, NK cell activation and migration caused an increase in IFN-γ production, which stimulates CXCL10 expression in cardiomyocytes and other resident myocardial cells in coxsackievirus B3 viral infection. This led to an inhibition of viral replication at an early stage in order to prevent cardiomyocyte damage and improve cardiac function [78].

Previous studies reported a synergy among double-stranded RNA (dsRNA) and IFN-γ-induced signaling in regulating CXCL10 production. As mentioned earlier, pro-inflammatory mediators and IFN-γ increased CXCL10 expression by a number of cell types, including keratinocytes, macrophages, endothelial cells, smooth muscle cells, and epithelial cell fibroblasts and astrocytes [79,80,81,82,83,84,85,86,87,88]. Majumder et al. reported that IFN-γ and TNF-α could promote CXCL10 production in human fibrosarcoma cell lines via binding the p48 complexes and the signal transducer and activator of transcription 1 (STAT-1) to ISRE site in CXCL10 promoter [89]. In another study, Oslund et al. determined that IFN-γ and influenza virus were able to synergistically induce CXCL10 in human airway epithelial cells through dsRNA-induced signaling both in vitro and in vivo [90]. Furthermore, the 735 bp proximal region of the CXCL10 promoter was the cause of this induction as it contains ISRE and NF-κB transcription factor binding sites. Following influenza virus infection, CD8^+^ T cell and NK cell migration towards the airway epithelial milieu was significantly affected by the ability of the airway epithelial cells to dramatically upregulate CXCL10 [90]. Other dsRNA-mediated pathways have also been identified in the upregulation of CXCL10, including retinoic acid-inducible gene I (RIG-I) and protein kinase R (PKR), which further augment the anti-viral signal by the activation of type I IFN production [91,92,93]. 

CXCL10 was found to have a protective function during certain RNA viral infections such as the severe acute respiratory syndrome coronavirus (SARS-CoV) and Epstein-Barr virus (EBV) [94,95,96]. On the other hand, CXCL10 was found to promote the infection caused by the human immunodeficiency virus (HIV) by stimulating the virus replication in macrophages and lymphocytes [97]. In short, the expression of CXCL10 was differentially associated with clinical symptoms in multiple infections. For example, a high expression of CXCL10 occurred before the development of clinical symptoms during HIV infection and murine retroviruses [98], and CXCL10 levels were positively correlated with organ damage and pathogen burden in HCV and HIV infections [99,100]. Moreover, persistent high levels of CXCL10 were linked to the failure of highly active antiretroviral therapy (HAART) in HIV-infected patients [101], as anti-retroviral treatment decreased the plasma levels of CXCL10, indicating its vital role in disease pathogenesis [97]. Additionally, the elevation of CXCL10 levels was reported for at least two weeks after disease onset, while corticosteroid therapy caused a reduction in the plasma CXCL10 levels, during SARS infection [102,103]. The chemokine receptor CXCR3 was highly expressed on viral-specific stem-like CD8^+^ T cells. CXCL10 regulates the persistence and heterogeneity of CD8^+^ T cells in the spleens of mice infected with lymphocytic choriomeningitis virus (LCMV). Moreover, functional CD8^+^ T cell responses were found to be greater in *Cxcl10*−/− mice and were associated with a lower viral count [104]. 

Among various chemokines, CXCR3 has been studied in viral hepatitis as it plays a significant role in the recruitment of T cells into the peripheral inflammatory sites [105,106]. The RNA virus, hepatitis A virus (HAV), is known to cause severe liver injury accompanied by the release of inflammatory mediators. HAV-infected hepatocytes produced multiple chemokines, including CXCL10. Moreover, CXCL10 levels were significantly increased in the serum of patients infected with HAV [107]. The CXCL10 production was reduced subsequent to inhibiting the signaling molecules associated with RIG-I-like receptor (RLR), such as mitochondrial antiviral signaling protein and interferon regulatory factor 3 (IRF3), in HAV-infected cells [107]. Similar to HAV, HCV-infected cells produced CXCL10 independent of type I or III IFNs [108], regulated by the expression of interferon regulatory factor 3 (IRF3) and NF-κB [109]. Additionally, CXCR3 is involved in the recruitment of effector CD8^+^ T cells and CD4^+^ T helper cells into HCV-infected livers [110,111,112], and the expression of CXCR3 in the liver or the peripheral blood is correlated with the severity of hepatic inflammation in HCV infection [113,114,115,116]. Similarly, the Kernow-p6 hepatitis E virus (HEV) strain was reported to stimulate the release of CXCL10 in enterocytes and infected hepatocytes [117,118,119,120]. Further, patients with HEV genotype three infections (clade efg) suffer from severe clinical representations, accompanied by higher CXCL10 serum levels and liver necro-inflammatory activity [121].

In chronic HCV infection, CXCL10 contributed to the development of necroinflammation and liver fibrosis. Moreover, the intrahepatic production of IFN-γ promoted CXCL10 expression by sinusoidal endothelium and hepatocytes, thereby triggering the recruitment of CXCR3^+^ T cells. CXCL10 plasma levels were significantly higher in HCV-infected patients with advanced fibrosis [122]. Additionally, CXCL10 levels were associated with the extrahepatic manifestations observed with this viral infection. For instance, CXCL10 levels were much higher in patients with HCV-associated cryoglobulinemia compared with those patients with autoimmune thyroiditis [122]. Additionally, high CXCL10 levels present in cases of HCV-associated cryoglobulinemia were associated with the presence of another extrahepatic manifestation, i.e., active vasculitis. Therefore, targeting CXCL10 and its receptor CXCR3 was suggested for treating HCV patients. Examples of such an approach include antibodies blocking the interaction between CXCR3 and its ligands, which reduced chronic liver inflammation and damage via the impairment of the infiltration of the nonspecific T cells [123]. This therapeutic strategy could be evaluated primarily for the non-responders to current anti-HCV therapy. This and other studies highlighted the potential use of CXCL10 as a prognostic marker of HCV clearance and successful therapy [122].

CXCL10 is also known to play a critical role in the host defense against infection caused by the John Howard Mueller (JHM) strain of mouse hepatitis virus (JHMV). Early expression of CXCL10 was observed to promote the attraction of CXCR3^+^ T cells into the central nervous system, which subsequently aids in limiting viral replication [124]. On the other hand, chronic CXCL10 expression contributed to the neuroinflammation and demyelination associated with JHMV infection. This was attributed to the attraction of CD4^+^ T cells that amplify neuroinflammation through the IFN induction of chemokine release. Therefore, the CXCL10/CXCR3 signaling pathway may influence glial biology and may repair virally induced neurologic diseases [124].

CXCL10 is a key player in respiratory syndromes. Its level was higher in the plasma and bronchial alveolar lavage fluid (BALF), which correlated with the disease severity [125,126,127]. Elevated CXCL10 levels were reported in patients with SARS or Middle East respiratory syndrome (MERS) as early as 2–3 days post-infection [61,103,128,129,130,131]. This increase was found to reduce the proliferation of myeloid progenitor cells, triggering lymphopenia as observed in coronavirus disease caused by severe acute respiratory syndrome coronavirus 2 (SARS-CoV-2) infection [131]. Additionally, CXCL10 was reported to trigger T cells apoptosis and lymphopenia seen in SARS-CoV-, MERS-, and SARS-CoV-2-infected patients, leading to T lymphocyte malfunction and worsening clinical outcome [132]. Previous studies using ex vivo human lung tissue explants demonstrated that the inoculation of SARS-CoV and SARS-CoV-2 induced the expression of various chemokines and cytokines, particularly CXCL10 [133]. 

Similar to other respiratory diseases, high CXCL10 levels in COVID-19 patients showed a strong correlation with disease severity [134,135]. As mentioned earlier, CXCL10 levels were increased in SARS-CoV-2 infected patients, especially those with acute respiratory distress syndrome (ARDS) [136,137]. Consequently, the use of CXCL10 as an independent predictor for COVID-19 progression was suggested since it was correlated with ARDS in critically ill patients and severe cases [132,138,139]. This was further supported in COVID-19 patients requiring intensive care unit (ICU) admission, who exhibited higher levels of CXCL10 than patients with mild infection [138,140,141,142,143]. Furthermore, higher mortality was reported in COVID-19 patients with a dramatic increase in CXCL10 levels compared with severe and mild COVID-19 patients [136,144,145,146]. In this regard, symptomatic COVID-19 patients showed higher levels of CXCL10 compared with convalescent cases [134]. Interestingly, CXCL10 levels were reported to be significantly reduced upon improving clinical outcomes in hospitalized COVID-19 patients [137]. Consequently, several studies reported CXCL10 as an excellent predictive biomarker of patient outcomes in COVID-19 [137,141]. CXCL10 was also identified to be a potential biomarker to predict left ventricular dysfunction in multisystemic inflammatory syndrome (MIS-C) patients, occurring post-exposure to COVID-19 infection [147].

Intriguingly, across all three coronaviruses (SARS-CoV, MERS-CoV, and SARS-CoV-2), CXCL10 was identified as a crucial contributor to pulmonary pathogenesis. CXCL10 was associated with lung injury and the activation of the toll-like receptor 4 (TLR4) signaling pathway [148,149]. Although not an ELR chemokine, CXCL10 induced neutrophil infiltration into the lungs, triggering further production of this chemokine, coupled with the release of oxidative burst by neutrophils. Eventually, this worsens the inflammatory lung state, causing progression to ARDS [150,151]. Therefore, antibodies against CXCL10 could be a potential and promising therapeutic approach in ARDS, as previously described in the influenza A virus subtype (H1N1) mouse model [150,152]. Furthermore, corticosteroids, the anti-inflammatory agents used in COVID-19 infection, were found to reduce the levels of multiple chemokines, including CXCL10, along with stimulating the NF-κB and activator protein-1 (AP-1) signaling pathways [153].

IL-6 is one of the cytokines released during COVID-19-mediated cytokine storm [154,155]. During this storm, high IL-6 levels act on lung endothelial cells, causing an increase in their permeability for serum proteins and the infiltration of inflammatory cells. This leads to an uncontrolled excessive immune response, such as in severe COVID-19 cases [154,155]. In addition, CXCL10 was recognized as a fundamental chemokine acting as a chemoattractant for monocytes, macrophages, and dendritic cells, as well as NK and T cells during COVID-19 infection. Upon the entry of SARS-CoV-2 into the lung epithelium, it triggers cytokine and chemokine production, which include IL-6 and CXCL10. One of the causes for the increased production of CXCL10 is IL-6, which also leads to an increase in the infiltration of CXCR3^+^ macrophages, the main producers of IL-6. These observations highlight the vicious cycle between CXCL10, IL-6, and macrophages in the lungs of COVID-19 patients, which could regulate the onset, maintenance, and progression of cytokine storms during COVID-19 infection [156].

As in respiratory coronavirus infections, CXCL10 showed a significant increase during measles virus (MeV) infection, which may distinguish MeV from other diseases such as rubella virus (RuV), parvovirus B19 (B19V), human herpesvirus 6 (HHV6), EBV, and human cytomegalovirus (HCMV). Another common factor was the high level of CXCL10 in hospitalized MeV patients compared with non-hospitalized ones, and higher CXCL10 level was observed in cases of primary infection compared with re-infected cases [157,158]. This demonstrates persistent inflammation and robust viral replication that might aggravate the clinical course of primary infection [159,160]. Interestingly, CXCL10 levels were associated with the serological stages of MeV infection. For instance, CXCL10 production was increased early after infection and reached its peak with the appearance of MeV-specific IgM antibodies, which later declined [157]. Another interesting finding during MeV infection is the association between mortality due to MeV infection and high CXCL10 levels in children. This further supports the link between CXCL10 and the clinical severity of MeV infection. CXCL10 and CCL5 were highly expressed in vitro and in vivo during RSV infection. However, CXCL10 was found to have a protective role by decreasing viral load and disease pathogenesis [161]. This was mediated via the promotion of leukocyte recruitment and the trafficking of dendritic cells (DCs). Furthermore, CXCL10 is stimulated by type I IFN in CXCR3^+^ DCs, triggering myeloid DC maturation [161]. 

**Figure 1:**
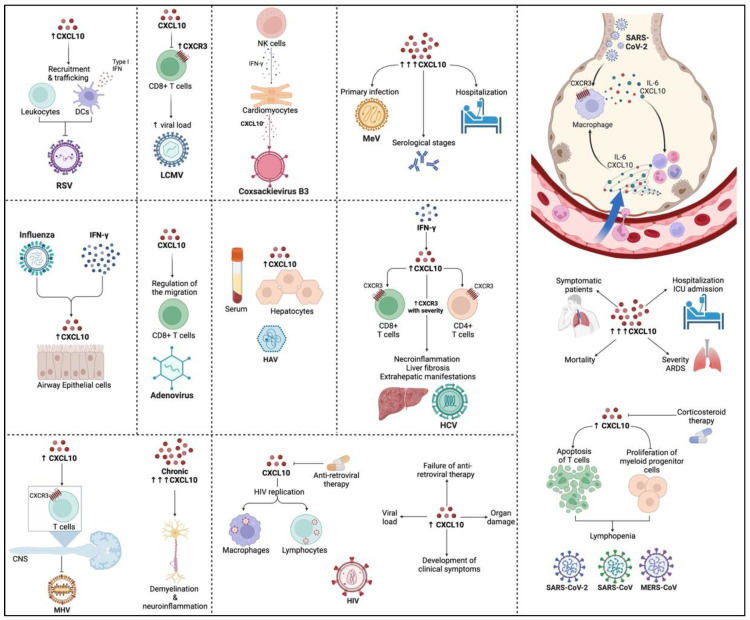
Role of CXCL10 during RNA viral infections. ARDS: acute respiratory distress syndrome, CNS: central nervous system, HAV: hepatitis A virus, HCV: hepatitis C virus, HIV: human immunodeficiency virus, LCMV: lymphocytic choriomeningitis virus, MERS-CoV: middle east respiratory syndrome coronavirus, MeV: measles virus, MHV: murine hepatitis virus, RSV: respiratory syncytial virus, SARS-CoV: severe acute respiratory syndrome coronavirus, SARS-CoV-2: severe acute respiratory syndrome coronavirus 2.

CXCL10 expression was higher in Zika virus (ZIKV)-infected individuals. This expression could be mediated through the induction of IFN-γ signaling pathway by the ZIKV NS5 protein. Previously, CXCL10 was discovered to be a biomarker of several bacterial infections [162]. Similarly, CXCL10 was identified as a biomarker of acute ZIKV infection and a predictor of disease severity [163]. A summary of the role of CXCL10 in RNA viral infections is shown in Figure 1.

## 4. Effects of CXCL10 during DNA Viral Infections

**Figure 2:**
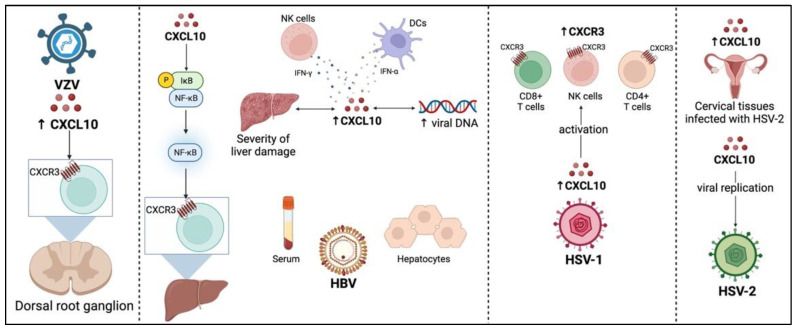
Role of CXCL10 during DNA viral infections. HBV: hepatitis B virus, HSV: herpes simplex virus, VZV: varicella-zoster virus.

As illustrated in Figure 2, CXCL10 is implicated in various DNA viral infections. For instance, CXCL10 was found to be a critical player in the innate defense against vaccinia virus infection by recruiting and activating NK cells [75]. In varicella-zoster virus (VZV) infection, upregulated CXCL10 expression was demonstrated along with the infiltration of CXCR3^+^ cells towards the infected dorsal root ganglia. Consequently, CXCL10 could be a distinct chemokine that attracts inflammatory cells, specifically CXCR3^+^ cells causing ganglionitis [164].

According to several studies, CXCL10 is implicated in hepatitis B virus (HBV) infection, including a study where the polymorphism G-201A in the promoter of the CXCL10 gene predicted susceptibility to chronic HBV infection [52]. During chronic infection, serum and intrahepatic levels of CXCL10 were elevated and correlated with HBV DNA and alanine aminotransferase (ALT) enzyme levels, coupled with progressive liver disease [165,166]. Other studies confirmed that CXCR3 ligands such as CXCL9, CXCL10, and CXCL11 are highly increased along with ALT in acute hepatitis patients, resulting from the release of IFN-α and IFN-γ by plasmacytoid DCs and NK cells, respectively, in response to HBV infection [167,168]. Furthermore, these three chemokines were decreased with reduced HBV DNA during acute infection [169]. In contrast, high CXCL10/IP-10 and CXCL9/Mig levels during HBV infection were correlated with severe liver disease. Subsequently, their neutralization was linked to the preservation of antiviral effects including less tissue damage [170]. Zhou et al. demonstrated that the upregulation of CXCL10 was dose-dependent through the activation of NF-κB, thereby enhancing the infiltration of peripheral leukocytes into the liver of HBV infection [171]. Consequently, blocking CXCL10 could reduce the recruitment of lymphocytes and improve the disease severity [71,172]. In HIV and HBV co-infected patients, mRNA levels for CXCL10 and CXCR3 were correlated with liver fibrosis. It has been suggested that the HIV infection of hepatocytes in the presence of HBV could promote inflammation and flux of CXCL10 production, thus promoting the recruitment of activated CXCR3^+^ cells towards the liver that could cause liver fibrosis [173]. 

CXCL10 is crucial in host defense against multiple neurotropic viruses, including herpes simplex virus-1 (HSV-1) [174]. It was previously reported that lack of either CXCL10 or its receptor CXCR3 could impair the mobilization of functional CD8^+^ effector memory (T_EM_) and CD8^+^ residual memory (T_RM_) cells within latently infected trigeminal ganglia following HSV-1 reactivation [175]. In contrast, increasing the levels of CXCL10 in latently HSV-1 infected CXCL10-deficient mice significantly restored the number of local antiviral CD8^+^ T_EM_ and CD8^+^ T_RM_ cells associated with protection against recurrent ocular herpes. These findings demonstrate that CXCL10/CXCR3 axis is crucial for CD8^+^ T cell immunity, which protects against recurrent herpesvirus infection [175]. The chemokine receptor CXCR3 is highly expressed on activated CD4^+^, CD8^+^ T cells, and NK cells at the sites of HSV-1 replication [176,177,178,179,180]. Additionally, during HSV-1 infection, CXCL10 was one of the first and most abundantly expressed chemokines, indicating that it may be essential for the coordinated immune response against HSV-1. Further, HSV-1 viral burden was elevated in CXCR3-deficient (CXCR3^−/−^) and CXCL10-deficient (CXCL10^−/−^) animals compared with wild types [174,177]. In this regard, CXCL10^−/−^ mice exhibited impaired NK and HSV-1-specific CD8^+^ T cell activation, which increased their susceptibility to HSV-1 infection [174]. 

Unlike HSV-1, CXCL10 was found to promote the infection caused by stimulating virus replication in immune cells during herpes simplex virus type 2 (HSV-2) infection [181]. The expression of CXCL9 and CXCL10 was increased in the cervical tissues of mice infected with HSV-2 [182,183,184]. At the same time, other studies reported an upregulation of CXCL10 and CXCL11 during HSV-2 infection [182,185,186]. Moreover, the HSV-2 protein infected-cell polypeptide 4 (ICP4) was suggested to be the critical viral component that stimulated the production of CXCL9, CXCL10, and CXCL11 [186]. 

## 5. Role of CXCL10 in Oncolytic Viruses

Multiple strains of oncolytic viruses such as vesicular stomatitis virus (VSV), HSV-2, maraba virus, chikungunya virus, and reolysin have been reported to drive the high expression of chemokines in infected tumors [187,188,189,190]. For instance, the intratumoral administration of oncolytic HSV-2 was associated with high CXCL10 expression, promoting the attraction of adoptively transferred T cells towards treated lesions [190]. Among many other cytokines and chemokines, CXCL10 was tested as an immune stimulant in the genomes of oncolytic viruses, to enhance the anti-tumor activity in several cancer studies [187]. Moreover, VSV was identified in promoting inflammatory tumor cell killing via the release of chemokines, including CXCL10, which trigger the infiltration of T effector cells into the tumor growth sites [191]. Another example was the oncolytic adenovirus with an inserted CXCL10 gene (Adv-CXCL10), which enhanced the recruitment of CXCR3^+^ T cells into the colon tumor microenvironment corroborated with increasing the efficacy of PD-1 antibody [192]. These results were translated into a clinical trial using an oncolytic adenovirus (NG-641) expressing CXCL9, CXCL10, and IFN-α, which is currently under investigation (NCT04053283) [193,194]. 

## 6. Conclusions

An impaired immune response is a critical hallmark in the development and progression of viral infections. Notably, the role of chemokines during viral infections has been explored and analyzed during the context of many RNA and DNA viral infections. CXCL10 was found to mediate its inflammatory activity through CXCR3, which is mainly expressed on T cells, NK cells, macrophages, and dendritic cells. Several studies report the significance of CXCL10 and its chemokine receptor CXCR3 in host defense and viral clearance, while other reports demonstrate its role in disease pathogenesis. Moreover, CXCL10 was suggested to be a potential diagnostic and prognostic marker in several viral infections such as COVID-19. Further, CXCL10 has a potential role as an anti-tumor agent mediated by oncolytic viruses. Therefore, targeting CXCL10 could be a possible therapeutic modality against many viral infections, as well as against tumor development. Further in-depth understanding of the chemokine system may allow the development of such therapeutics against viral infections, which could be beneficial in preventing opportunistic microbes.
